# Traditional healer support to improve HIV viral suppression in rural Uganda (Omuyambi): study protocol for a cluster randomized hybrid effectiveness-implementation trial

**DOI:** 10.1186/s13063-024-08286-4

**Published:** 2024-07-01

**Authors:** Radhika Sundararajan, Misha Hooda, Yifan Lai, Denis Nansera, Carolyn Audet, Jennifer Downs, Myung Hee Lee, Margaret McNairy, Winnie Muyindike, Juliet Mwanga-Amumpaire

**Affiliations:** 1 Weill Cornell Medicine, 1300 York Ave, New York, NY 10065 USA; 2 Mbarara University of Science and Technology, Mbarara, Uganda; 3 Vanderbilt University Medical Center, 1211 Medical Center Dr, Nashville, TN 37232 USA

**Keywords:** HIV care, Traditional healers, HIV viral load, Implementation science, Protocol, Community-based intervention

## Abstract

**Background:**

Rural African people living with HIV face significant challenges in entering and remaining in HIV care. In rural Uganda, for example, there is a threefold higher prevalence of HIV compared to the national average and lower engagement throughout the HIV continuum of care. There is an urgent need for appropriate interventions to improve entry and retention in HIV care for rural Ugandans with HIV. Though many adults living with HIV in rural areas prioritize seeking care services from traditional healers over formal clinical services, healers have not been integrated into HIV care programs. The Omuyambi trial is investigating the effectiveness of psychosocial support delivered by traditional healers as an adjunct to standard HIV care versus standard clinic-based HIV care alone. Additionally, we are evaluating the implementation process and outcomes, following the Consolidated Framework for Implementation Research.

**Methods:**

This cluster randomized hybrid type 1 effectiveness-implementation trial will be conducted among 44 traditional healers in two districts of southwestern Uganda. Healers were randomized 1:1 into study arms, where healers in the intervention arm will provide 12 months of psychosocial support to adults with unsuppressed HIV viral loads receiving care at their practices. A total of 650 adults with unsuppressed HIV viral loads will be recruited from healer clusters in the Mbarara and Rwampara districts. The primary study outcome is HIV viral load measured at 12 months after enrollment, which will be analyzed by intention-to-treat. Secondary clinical outcome measures include (re)initiation of HIV care, antiretroviral therapy adherence, and retention in care. The implementation outcomes of adoption, fidelity, appropriateness, and acceptability will be evaluated through key informant interviews and structured surveys at baseline, 3, 9, 12, and 24 months. Sustainability will be measured through HIV viral load measurements at 24 months following enrollment.

**Discussion:**

The Omuyambi trial is evaluating an approach that could improve HIV outcomes by incorporating previously overlooked community lay supporters into the HIV cascade of care. These findings could provide effectiveness and implementation evidence to guide the development of policies and programs aimed at improving HIV outcomes in rural Uganda and other countries where healers play an essential role in community health.

**Trial registration:**

ClinicalTrials.gov NCT05943548. Registered on July 5, 2023. The current protocol version is 4.0 (September 29, 2023).

**Supplementary Information:**

The online version contains supplementary material available at 10.1186/s13063-024-08286-4.

## Background

In Uganda—as in most African countries—the majority of the population resides in rural communities [1]. Rural areas bear a disproportionate burden of HIV; in Uganda, there is a nearly threefold increase in the prevalence of HIV in some rural areas compared to the national level [2]. According to the most recent Ugandan population-based HIV impact assessment, only 66% of rural people living with HIV (PLWH) are aware of their status, compared to 86% of urban PLWH. Furthermore, only 39% of rural PLWH are on antiretroviral therapy (ART), and 23% are virally suppressed, compared to 75% and 59% of urban PLWH, respectively [3]. Overall, rural populations demonstrate 15–25% lower engagement in the HIV continuum than their urban counterparts [4]. These data indicate a pressing need for appropriate and effective strategies to improve rural HIV care to achieve the UNAIDS 95–95-95 benchmarks [5].

In rural areas, PLWH encounter many challenges accessing and consistently engaging in HIV care. Clinics are few and far between, often necessitating expensive motorized transportation. Consequently, regular clinic visits are challenging for individuals living in poverty [6–8]. Moreover, a considerable number of rural PLWH harbor mistrust toward medical care, stemming from fears of stigma associated with being spotted at an HIV clinic, concerns over breaches in confidentiality, and perceived mistreatment from clinicians [9–12]. A lack of social support and diminished self-efficacy among these populations further contribute to disinterest and disengagement from HIV care [13, 14]. Therefore, community-based strategies designed to overcome these obstacles will likely be particularly beneficial for rural PLWH.

Decentralized HIV care mitigates barriers for rural populations by shifting support mechanisms to communities where PLWH reside. The strategy of training community lay providers to offer nonclinical support, such as psychosocial guidance and adherence support, has effectively increased entry and retention in the HIV continuum of care throughout HIV-endemic global settings [15–20]. Consequently, decentralized care has been recognized and integrated into AIDS control strategies by major health organizations, including the World Health Organization (WHO), the Ugandan Ministry of Health, and the President’s Emergency Plan for AIDS Relief (PEPFAR) [17, 21, 22]. Existing community-based programs, however, primarily involve community health workers, village health teams, and peers of PLWH. Traditional healers (TH) largely remain untapped resources as community lay supporters. This fact is a particularly relevant oversight given that > 80% of African populations routinely use TH—both as an adjunct to, or instead of, formal biomedical care. TH are often the first point of contact for healthcare services in their communities, and many adults, including PLWH, may preferentially visit TH because of their accessibility and ubiquity in the region [23, 24]. Therefore, leveraging TH to provide HIV support to their clients provides a potential opportunity to improve HIV outcomes in rural communities [9, 25–27].

The Omuyambi trial aims to evaluate the effectiveness of TH-delivered nonclinical support for adults with unsuppressed HIV viral loads in rural Uganda. This trial adopts an implementation science approach to evaluate contextual determinants, facilitators, and barriers to implementing this novel program. Our central hypothesis is that training TH to engage and support rural PLWH will improve HIV viral load suppression compared with standard care. This study has two objectives:

1. Determine the effectiveness of TH-delivered support (intervention) on HIV viral suppression versus standard clinic-based HIV care alone (control) in a cluster randomized trial. Secondary clinical outcomes include (re)initiation of HIV care, ART adherence, and retention in care.
2. Evaluate the context, facilitators, and barriers pertaining to the implementation of the TH-delivered program. This evaluation will be guided by the Consolidated Framework for Implementation Research (CFIR) domains of intervention characteristics, individuals involved, inner setting, outer setting, and implementation process [28–31]. Implementation outcomes will include adoption, acceptability, appropriateness, fidelity, and sustainability.

### Evidence-based program selection and adaptation

Prior to study initiation, the Omuyambi study team identified an evidence-based lay counseling program that could be adapted for TH delivery. We selected the Patient Advocate Program to adapt to rural Ugandan healers. This community health worker-delivered intervention was developed for PLWH who defaulted from care and assists in (re)linking PLWH to HIV care, supporting ART (re)initiation and adherence through medication counseling, and facilitating retention in care through the provision of individual psychosocial support. A multicenter cohort study among ~ 67,000 PLWH at 57 sites in South Africa showed that the Patient Advocate Program improved viral suppression 8 years after ART initiation compared with routine care (88.6 vs. 80.6%, adjusted RR = 1.53) [32, 33]. This evidence-based program required adaptation for our setting, as it was developed in a semiurban South African context with a robust force of community health workers, unlike rural Uganda. We adapted the program for TH as they are accessible and trusted lay providers in rural Ugandan communities where health facilities are scarcer, literacy is lower, and baseline viral suppression is poorer than the original intervention location. Details of the adaptation process and pilot testing of the TH-delivered program can be found in Sundararajan et al. [34].

## Methods

### Study design

**Fig. 1:**
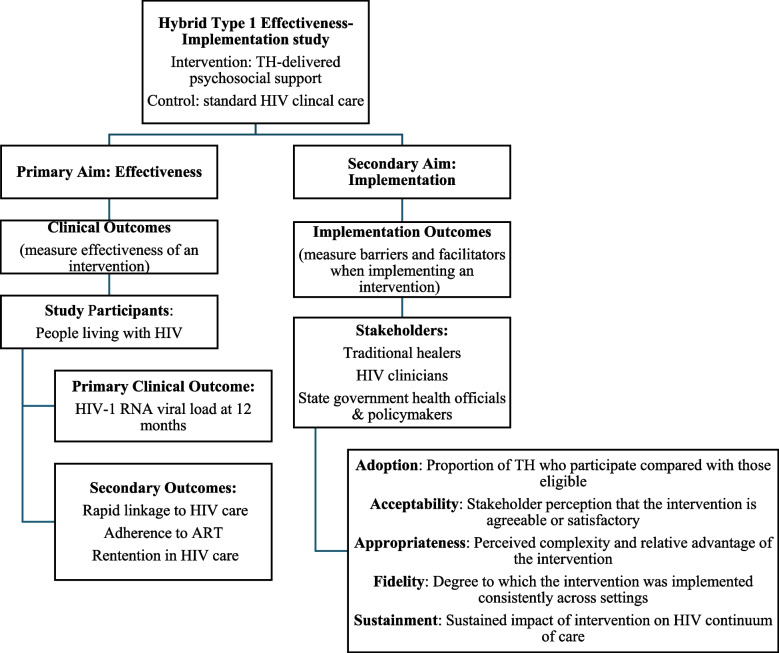
Omuyambi clinical trial flowchart

This is a hybrid type I effectiveness-implementation study, where the effectiveness of an intervention is evaluated while concurrently collecting data on program implementation. This effectiveness study is a parallel-arm, cluster randomized trial that will assess the impact of TH-delivered psychosocial support as an adjunct to standard care (intervention) versus standard HIV clinic-based care alone (control) on HIV viral load. The unit of randomization in this study is the individual TH. TH randomized to the intervention arm will provide psychosocial support to PLWH with unsuppressed HIV viral loads who receive traditional care at their practices. Intervention effectiveness will be measured through the primary outcome of HIV viral load suppression at 12 months after enrollment, with secondary clinical outcomes that include (re)initiation in HIV care, ART adherence, and retention in care. Implementation will be evaluated via in-depth interviews and structured surveys among key stakeholders (TH, PLWH, clinicians, and health officials from the Ministry of Health AIDS Control Division Officers and District Health Offices). Implementation barriers and facilitators will be identified, in addition to outcomes such as intervention adoption, fidelity, acceptability, appropriateness, and sustainment. The Omuyambi clinical trial flowchart (Fig. 1) provides a summary of the study design and outcomes. The SPIRIT checklist was used to guide protocol development (Additional File 1) [35].

### Study setting

**Fig. 2:**
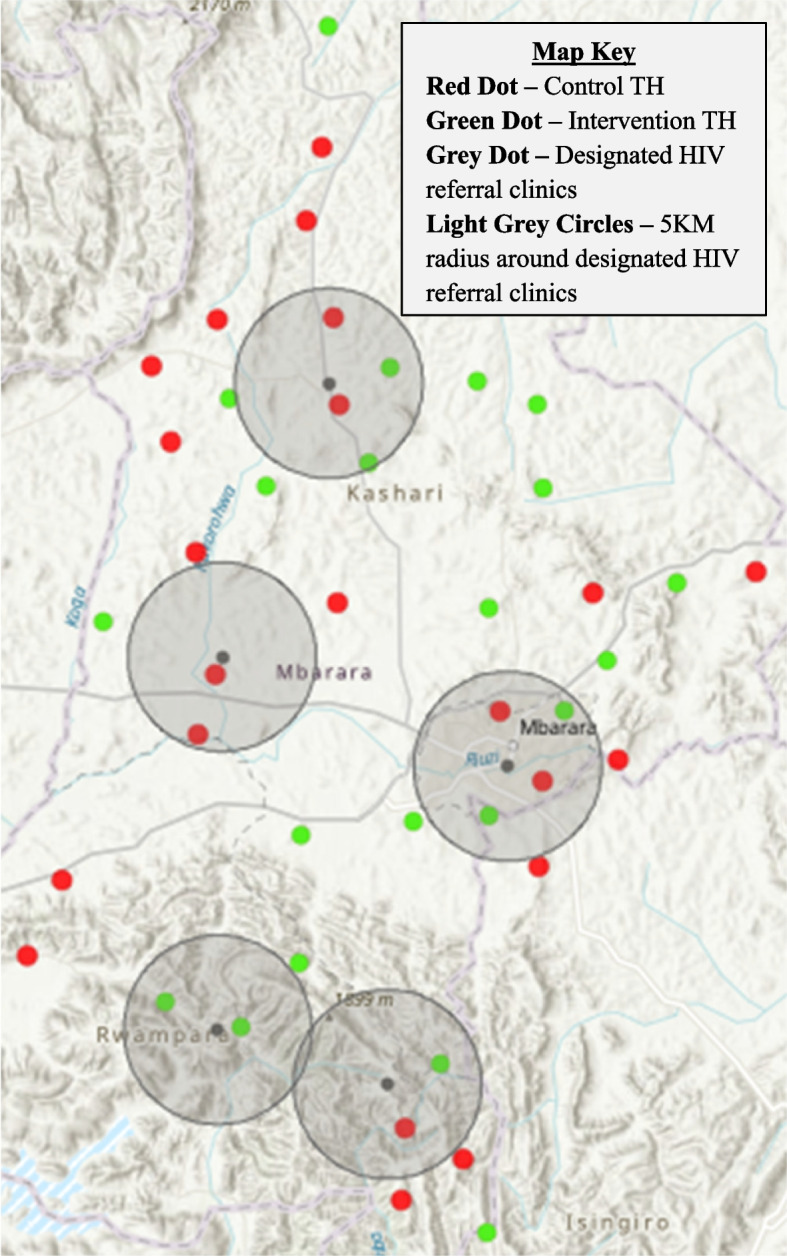
Geographical map of the 5 designated HIV referral clinics and 44 enrolled TH locations

This trial will be conducted in two rural districts of southwestern Uganda (Mbarara and Rwampara). These study regions are ~ 250 km from the capital, with a population of 480,000 across 1780 km^2^ [36]. The prevalence of HIV is 8% in the Mbarara and Rwampara districts, exceeding the national prevalence of 6% [3]. HIV care in the study region is provided at no cost in 15 government-run hospitals and health centers. Five government HIV clinics within the study region were selected to serve as referral sites for the study. These represent the five clinics where most of the PLWH in our prior studies in the region received care. Forty-four eligible TH were identified and then randomized to cluster sites (please see the “Study population and eligibility criteria” and “Randomization” sections below for details). PLWH will be recruited from these TH clusters. Figure 2 depicts a geographical map of the 5 designated HIV referral clinics and 44 enrolled TH locations. For implementation evaluations, clinicians will be recruited from referral HIV clinics, and health officials will be recruited from the Ministry of Health and District Health offices.

### Study population and eligibility criteria

#### Inclusion criteria

##### For PLWH

1. Positive point-of-care HIV Oraquick Test result or CASE Adherence Index Questionnaire [37] score ≤ 10
2. HIV viral load ≥ 200 copies/mL [38] at the time of enrollment
3. Age ≥ 18 years
4. Primary residence in Mbarara or Rwampara districts
5. Self-reports of not being in clinical care if previously diagnosed with HIV
6. Able to provide informed consent

##### For traditional healers

1. Monthly volume of ≥ 5 adult clients
2. Location ≥ 3 km from another participating TH cluster
3. Age between 18 and 75 years, inclusive
4. Agreements to attend all training/courses associated with their respective study arms
5. Able to provide informed consent

##### For HIV clinicians and officers from the Ministry of Health AIDS Control Division or District Health Office

1. Being employed in their position for ≥ 1 year
2. Aged 18 or older
3. Able to provide informed consent
4. Patient-facing roles (physician, nurse, social worker, counselor) in one of the referral clinics

Potential participants who do not meet the inclusion criteria are ineligible. There are no specific exclusions.

### Interventions

#### Procedures for both study arms

Prior to trial initiation, all 44 TH will participate in a day-long training led by HIV clinicians, which will include information on HIV transmission and prevention, pre- and post-HIV test counseling, and the use of oral swab self-testing kits. The training curriculum is shown in Table 1. Refresher training will take place at 6-month intervals after the initial training to reinforce these concepts and facilitate discussions between TH and clinicians.

**Table 1:** Omuyambi training curriculum

**Day 1 (both study arms)**
1. HIV/AIDS overview
a. Knowledge and epidemiology of HIV in Uganda
b. Rationale for ART use and adherence (U = U)
2. Ethics of working with PLWH
a. Confidentiality and status disclosure
b. Discrimination and stigma
3. Overview of clinical services for PLWH
a. Enrollment and pre-ART counseling
b. ART initiation
c. Treatment of other opportunistic infections
d. Adherence support and monitoring
4. Overview of study procedures
a. HIV pre- and post-test counseling
b. Use and interpretation of Oraquick HIV self-testing
c. Linking newly diagnosed patients to HIV care
**Day 2 (intervention only)**
1. Community resources for PLWH
a. NGO or government offices
b. Community support groups
c. Advocacy groups for stigma reduction
2. ART readiness, adherence barriers/facilitators
3. The role of lay adherence supporters
a. Supporting PLWH outside the clinic
b. Lay support versus clinical care roles
c. Lay supporter as advocate and confidante
4. ART adherence support strategies
a. Strengths-based counseling/self-efficacy
b. Managing ART side effects
c. Emphasizing goals of treatment
5. Communication skills for psychosocial counseling
**Day 3 (intervention only)**
1. Strategies for healthy/positive living
a. Nutrition, exercise, and mental health
b. Routine healthcare and clinic visit
c. Safer sex
2. HIV status disclosure and social support
a. Counseling on disclosure strategies
b. Improving social support
3. Identifying mental health symptoms
a. Introduction to depression and anxiety
b. Common symptoms and solutions
c. When to refer PLWH for further management

Our team previously demonstrated the feasibility and effectiveness of TH-facilitated rapid HIV testing [39]. As such, both intervention and control healers will identify new PLWH at their practices through oral-swab HIV self-testing kits (OraQuick ©). To be eligible to receive a self-testing kit, clients must be ≥ 18 years or older, report no prior HIV diagnosis, and have not had an HIV test in the past 12 months. TH in both study arms will also use the CASE adherence index to screen for disengagement from care and ART nonadherence among known PLWH [37]. Potentially eligible clients (newly diagnosed or with CASE adherence scores ≤ 10) will be consented by a study team member, and a venous blood sample will be collected to confirm study eligibility via an HIV viral load assay.

Eligible PLWH in both study arms will be referred to one of five government-run HIV clinics where they can receive free HIV care according to Ugandan MoH guidelines [17]. One clinician from each of the five referral clinics will serve as a clinical liaison and will be responsible for helping to arrange linkage to care appointments and tracking clinic attendance among PLWH participants. TH and PLWH participants will cooperatively select which of the five referral clinics best suits their needs. As the cost of transport to clinic appointments is a known barrier to accessing HIV care, all PLWH will be provided with funds to cover the cost of transportation to their first clinic appointment [40, 41].

#### Control arm study procedures

No additional linkage or psychosocial support will be delivered at the control arm cluster sites to PLWH other than the compensation provided for transportation to (re)establish HIV care. The control arm TH will receive monthly compensation for the time spent facilitating HIV self-testing, referring clients with reactive tests, and identifying PLWH who have defaulted from care. Additional phone voucher reimbursements will be provided to facilitate communication with the study team.

#### Intervention arm study procedures

The TH clusters randomized to the intervention arm will attend two additional days of training prior to the launch of the trial. These training sessions will focus on the principles and strategies for delivering psychosocial support to PLWH. For enrolled PLWH, intervention TH will actively facilitate linkage to one of the five predetermined referral clinics, with the goal of rapid linkage to care within 7 days, according to World Health Organization guidelines [42–44]. The PLWH have the option for the TH to accompany him/her to clinical appointments.

Over the following 12 months, TH will provide psychosocial support for PLWH via the TH-tailored curriculum as an adjunct to routine HIV care. TH will employ one-on-one counseling to improve self-efficacy, provide social support, and develop individualized adherence strategies for participating PLWH. Psychosocial support will be delivered through approximately 30-min meetings with the PLWH. TH will meet with PLWH weekly for 1 month. After that, meetings occur once a month unless TH determine a need for more frequent meetings. TH and PLWH can schedule phone calls when in-person meetings are not possible. TH will be encouraged to use a pragmatic approach to incorporate core elements of the intervention and tailor these components for individual PLWH. There will be no charges to clients for recurring TH visits associated with Omuyambi. The intervention TH will receive a monthly stipend as compensation for work as lay supporters, plus reimbursement in phone vouchers to facilitate communication with RAs, PLWH, and clinical liaisons.

### Trial outcomes

All trial outcomes are summarized in Table 2.

**Table 2:** Omuyambi trial outcomes

Domain	Measurement	Method of measurement	Outcome definition	Time point
**Primary clinical outcome**	HIV-1 RNA viral load in blood plasma samples	Abbott HIV-1 assay of venous blood sample	Plasma HIV-1 RNA < 200 copies/mL^2^	12 months following enrollment
**Secondary clinical outcomes**				
Rapid linkage to HIV care	PLWH attending a clinic visit within 7 days of study enrollment	Clinical liaison or HIV treatment card documentation	Completing first clinic visit within 7 days of enrollment	7 days following enrollment
Adherence to ART	Tenofovir concentration in hair samples	Hair sample analysis	Hair tenofovir concentration of > 0.023 ng/mg	12 months following enrollment
Retention in HIV care	PLWH attending most recent HIV clinical appointment	Clinical liaison or HIV treatment card documentation	Attending the most recent appointment within 90 days of its scheduled date	12 months following enrollment
**Implementation outcomes**				
Adoption	The proportion and representativeness of intervention agents who are willing to initiate a program	TH who agree to participate of the total number invited	Proportion of TH who participate compared with those eligible	Baseline
Acceptability	Theoretical Framework of Acceptability (TFA) scale and qualitative interviews	Structured survey and qualitative interviews with PLWH, TH, and clinicians	Perceived complexity and relative advantage of the intervention	Baseline, 12, and 24 months after study initiation
Appropriateness	Qualitative interviews	Interviews with PLWH, TH, and HIV clinicians	Perceived fit, relevance, and compatibility of the intervention for the study setting	Baseline, 12, and 24 months after study initiation
Fidelity	Observation checklist	Structured observations of intervention TH	Degree to which the intervention was implemented consistently across settings	Quarterly
Sustainment	HIV-1 RNA viral load in blood plasma samples	Abbott HIV-1 assay of venous blood sample	Sustained impact of intervention on HIV continuum of care	24 months after study initiation

#### Effectiveness outcomes

The primary study outcome is HIV viral suppression (serum viral load < 200 copies/mL) among PLWH at 12 months following study enrollment. Plasma samples will be processed to obtain HIV-1 RNA viral load concentrations at the Epicenter Mbarara Research Base using the Abbott HIV-1 assay. This test is > 90% sensitive and specific [45–47]. The secondary outcomes will be (1) (re)linkage to care within 7 days; (2) ART (re)initiation; (3) ART adherence at 12 months after linkage to care, defined as a binary outcome of hair tenofovir concentration (> 0.023 ng/mg, reflecting four or more ART doses per week); and (4) retention in HIV clinic care after 12 months [48, 49].

#### Implementation outcomes

The implementation outcomes assessed are guided by the Consolidated Framework for Implementation Research (CFIR) and include intervention adoption, acceptability, appropriateness, feasibility, fidelity, and sustainment evaluations [28, 50].

### Recruitment and informed consent

TH in the study area were identified through a door-to-door census of the study region conducted by the study team in June 2023. All TH meeting the eligibility criteria had their GPS location documented. Forty-four TH were randomly selected from this census for possible trial participation and invited to participate as cluster sites. All TH provided written informed consent.

The participating TH will identify eligible PLWH. For this study, TH will not recruit PLWH outside of their practice. TH will provide basic information about the study to potentially eligible participants, and if the participant is interested, the TH will ask them for permission to be contacted by the study team. Members of the study team will contact the eligible PLWH by mobile phone within 24 business hours to provide additional information about the study and confirm interest in participating. RAs will arrange for an in-person meeting at a convenient location to request written informed consent from the potential participant and collect a venous blood sample for HIV viral load testing. If the participant wishes to participate, the study staff member will have the participant sign and date the consent form in the space provided. If the PLWH has limited literacy, they will select someone to witness the entire consent process. If the participant is unable to sign or print their name or date, the witness will print the participant’s name and date, and the participant will use the ink pad to make their thumbprint. The witness will sign and date the consent form in the space provided. All questions from the study surveys and questionnaires will be delivered verbally to accommodate those with limited literacy.

For implementation evaluations, clinicians and policymakers will be recruited through purposive sampling through the study referral HIV clinics and local and Ministry of Health offices, respectively. They will provide written informed consent for participation.

Informed consent forms will be written in nontechnical language, translated from English into Runyankole (local language) and then translated back to English to ensure accuracy. The consent process will be conducted by trained study team members who are fluent in Runyankole and English. Study team members will all be required to maintain Good Clinical Practice certification and will be trained on the research protocol. Recruitment is targeted for completion by the end of July 2025. All study participants are volunteers and can leave the study at any time without recourse. This is clearly stated during the informed consent process.

### Sample size

Study power calculations are based on the primary hypothesis that the intervention will improve viral suppression by 20% using standardized methods for cluster randomized trials [51]. Based on population-level data from the Ugandan Ministry of Health, we assume that the proportion of PLWH achieving the primary outcome of viral suppression will be 60% in the control arm versus 80% in the intervention arm [3]. The results of prior community-based and psychosocial HIV support interventions were used to inform this estimated effect size [32, 52, 53]. To ensure 85% power to detect a difference of ≥ 20% in viral suppression between arms and assuming a 10% loss-to-follow-up rate, we will enroll a minimum of 16 PLWH in each cluster. The target sample size is 650 PLWH, with 325 PLWH in each arm. Forty-four TH will be enrolled as cluster sites, with 22 in each study arm.

As part of the implementation evaluation, we will purposively recruit 20 PLWH, 20 HIV clinicians, and 7 policymakers for participation. The sample size of 20 HIV clinicians is based on prior research showing that thematic and conceptual data saturation is obtained after 12–16 interviews have been conducted within a subgroup [54, 55]. Due to the limited number of policymakers, 7 interviews will be conducted to understand local and national government priorities and implementation context.

### Randomization

Among the TH in the region who met the study inclusion criteria, 44 were randomly selected to participate in the study. Eligible TH were assigned to a stratum corresponding with geographic distances either near (≤ 5 km) or far (> 5 km) from the study referral HIV clinics (Fig. 2). Five kilometers will be used to define strata as the median distance from TH in the study region to the nearest HIV clinic. Within each stratum, subgroups of TH were created to include one TH and any others located ≤ 3 km away from one another to minimize contamination between clusters.

Using computer-generated random assignment, the principal investigator randomly selected 44 TH from the subgroups. Using another computer-generated random assignment, each of the 44 selected TH was randomized at a 1:1 ratio within each stratum and assigned to either the intervention or control arm. Following randomization, TH were informed of their assigned study arm by a member of the study team and informed of the upcoming training requirements.

### Blinding

There will be no blinding in this study except during the data analysis. Due to the evident morphological differences between the intervention (psychosocial support) and control (no psychosocial support) groups, both participants and study team members will be aware of study arm allocations following randomization. The study statistician will be blinded to study arm allocations during analysis.

### Participant timeline

Please see Table 3 for the detailed schedules of activities for each participant group.

**Table 3:** Detailed schedules of activities for each participant group

People Living with HIV (PLWH) Schedule of Activities							
**Period/Phase**	**Screening/Baseline**	**Day 15**	**Day 29**^**d**^	**Day 43**^**d**^	**Day 57**^**d**^	**12-Month F/u**	**24-Month F/u**
**Study Day**	**D1**	**D15**	**D29**	**D43**	**D57**	**D365**	**D731**
**Study Week**	**W1**	**W2**	**W4**	**W6**	**W8**	**W52**	**W104**
**Study Visit**	**1**	**2**	**3**	**4**	**5**	**6**	**7**
**Window (Days)**		** ± 7**	** ± 7**	** ± 7**	** ± 7**	** + 30**	** + 30**
Oraquick HIV Testing^a^	x						
Case Adherence Index Questionnaire^b^	x						
Informed Consent	x						
Assessment of Inclusion/Exclusion Criteria	x						
Demographic Collection^c^	x						
Medical History	x						
HIV History	x						
FSSQ [56]	x					x	x
HIV-ASES [57]	x					x	x
HIV Stigma Questionnaire [58]	x					x	x
HIV-SMS [59]	x					x	x
HIV-KQ-18 [60]	x					x	x
Quality of Life (MOS-HIV) [61]	x					x	x
TFA [62]	x					x	x
Dried Blood Sampling						x	x
Hair Sample Collection						x	
Patient Enrollment card	x					x	x
**Interviews (will be conducted for specified patients)**	x					x	x
*Perceived complexity and relative advantage of the intervention*	x					x	
*Perceived successful strategies with implementers & patients*	x					x	
*Perceived roles of intervention champions & opinion leaders*	x					x	
*Facilitators & barriers to implementation*						x	
*Sustained impact of intervention on HIV continuum of care*							x
*Integration into routine practice; reasons for maintenance/discontinuation*							x
Phone Call to Patient about Linkage to HIV Care		x	x	x	x		

^a^Oraquick HIV Testing to be administered prior to study enrollment to adults reporting no prior HIV diagnosis or HIV testing within the prior 12 months

^b^Case Adherence Index Questionnaire to be administered prior to study enrollment and to suspected defaulting participants only

^c^Demographics include includes age, sex, education, religious affiliation, household size, marital status, highest level of education

^d^Visit only occurs if PLWH has not linked to care within 7 days

All PLWH and TH will meet for scheduled study visits with a member of the study team at enrollment (baseline) and then at 12 and 24 months following enrollment to complete structured surveys. Qualitative interviews will be held throughout the study period, beginning at the time of enrollment. We will invite all intervention arms TH and clinical liaisons, as well as a sample of PLWH, HIV clinicians, and policymakers, to participate in these interviews. Study participants may request to discontinue participation in the study at any time. If a study participant fails to attend their 12-month or 24-month research study visits, they will be considered lost to follow-up. All participant data will be analyzed under the principle of intention-to-treat.

### Participant retention

#### Retention of TH clusters

Upon enrollment, TH were assigned a unique identification number, and the study team collected detailed locator information, including contact information for the TH, multiple designated contact persons, and detailed residence information for all TH who provided informed consent to participate in the trial. We will provide contact information for our study team members to the TH and will remain available to answer any questions that arise. In the 2 weeks leading up to the biannual TH refresher trainings, an RA will call each participating TH to remind them of the upcoming training time and location. RAs will also send a reminder text message the day before the refresher trainings. If a TH is not reachable by phone, RAs will make site visits to remind the TH in person.

#### Retention of PLWH participants

Upon enrollment, study participants will be assigned a unique identification number and will complete a detailed locator information form that includes contact information for the participant, multiple participant-designated contact persons, and detailed residence and workplace information. These designated contact persons will be peers or family members whom the study team can contact if the participant is not reachable for follow-up through other means. Study staff will contact participating PLWH via mobile phone following enrollment to assess for (re)linkage to care within 7 days of study enrollment. For participants who cannot be reached by phone during the initial follow-up, study staff will call a backup contact to inquire about the participant’s whereabouts (taking care not to mention HIV status or the purpose of the study). If participants are not reachable via their designated contacts, study staff will invoke the assistance of participants’ TH to help locate the individual, as TH are often in regular contact with their clients. Participants will be notified 2 weeks by phone call prior to the study staff’s 12- and 24-month visits and receive a reminder the day before via text message.

#### Retention of HIV clinic staff and liaisons

Upon enrollment, clinic staff and liaisons will be assigned a unique identification number, and we will collect contact information for each clinician, including mobile number and workplace information. The study staff will contact clinicians 2 weeks ahead of schedule to arrange interviews at specific times and in private locations convenient for the clinicians. If clinicians are unavailable by phone, research assistants will visit their workplaces to schedule their interviews.

#### Retention of policymakers

A sample of seven health officials in the Ministry of Health AIDS Control Office or District Health Offices will be invited to participate in three individual interviews, the first at the time of the study launch, then again after 12 and 24 months. Upon enrollment, each health official will be assigned a unique identification number, and we will collect contact information for each official, including mobile number and workplace information. RAs will contact officials 2 weeks ahead of schedule to arrange interviews at specific times and private locations convenient for the officials.

### Data collection and management

#### Quantitative data collection

Study staff and clinical liaisons will collect data on the effectiveness of the intervention. PLWH participants will receive an in-person visit from a member of the study team to complete a plasma blood draw for the HIV-1 RNA viral load assay at baseline, 12 months, and 24 months, and a hair sample collection at 12 months following enrollment. All the data will be collected in private locations. Data regarding engagement with clinical care will be reported by liaisons or abstracted by the study team from clinic cards. All clinical data will be entered into a secure database (REDCap) hosted by Weill Cornell Medical College.

The study team will collect quantitative data on intervention implementation. All TH (*n* = 44) and PLWH (*n* = 650) will complete survey items administered by the study staff. Translated questions will be loaded onto encrypted study tablets, and responses will be entered directly into REDCap. RAs will read items aloud to accommodate participants with limited literacy. The Theoretical Framework of Acceptability (TFA) scale will be administered at baseline and 12 months [62–64]. Fidelity will be measured via quarterly structured observations, with intervention TH to be scored by a member of the study team according to core elements of the intervention curriculum: (1) facilitating rapid linkage to HIV care and (2) delivery of psychosocial support using one or more key elements (ART adherence, healthy living, HIV status disclosure, identifying mental health symptoms) [65]. Adoption will be quantified as the number of eligible TH invited who agree to participate as cluster sites. Reasons for refusal to participate will also be documented. Finally, maintenance will be assessed 12 months after training and support for the intervention has ended. We will quantify the maintenance of viral suppression via HIV-1 viral load measurements from PLWH in both study arms 24 months after enrollment.

Additional participant data collected at enrollment will include age, sex, income, education attained, religious affiliation, marital status, and history of HIV testing. PLWH quality of life will be measured using the Medical Outcomes Study-HIV Health Survey (MOS-HIV) [66]. PLWH, TH, and clinician HIV knowledge will be measured using the HIV Knowledge Questionnaire (HIV-KQ-18) [60]. PLWH social support will be measured using the Duke-UNC Functional Social Support Questionnaire (FSSQ) [67]. PLWH self-efficacy will be measured by the HIV-ASES scale [57]. PLWH, TH, and clinician stigma will be measured using the HIV-Stigma Scale [58]. Furthermore, PLWH stigma will also be measured using the HIV Stigma Mechanisms Scale (HIV-SMS), and TH and clinician stigma will be measured using the HIV Stigma and Discrimination Scale [59, 68].

#### Qualitative data collection

Overall, 69 participants will be invited for qualitative interviews (*n* = 22 intervention TH, *n* = 20 intervention PLWH, *n* = 20 HIV clinicians, and 7 health officials). TH, PLWH, HIV clinicians, and health officials will participate in three interviews (at baseline, 12 months after study initiation, and 24 months after study initiation). Participants will be purposively sampled to share first-hand knowledge about their experiences with intervention [14]. We will select interview participants to ensure representation by sex, age, clinical position (for HIV clinicians), geographic distribution, and viral load (for PLWH).

The interviews will be guided by CFIR as a framework (Table 2), conducted in private locations for ~ 60 min, and based on an interview guide to allow for the exploration of novel topics and the elicitation of information-rich data [28–31]. The interview guides will be composed in English, translated into Runyankole, and translated back to English to ensure the integrity of meaning. Interviews in Runyankole will be conducted by trained members of the Ugandan qualitative team, audio-recorded and transcribed and translated into English by the interviewer. Transcripts will be spot-checked against audio recordings by the Qualitative Data Coordinator and the Ugandan PI (both fluent in Runyankole and English) to ensure translation validity and integrity.

### Data management and confidentiality

All quantitative study data will be collected using Android tablets and uploaded daily into the REDCap data management system, which is compliant with Good Clinical Practice and 21 CFR Part 11 regulations [69, 70]. All de-identified qualitative study data will be uploaded to a secure OneDrive, which is password-protected and compliant with NIH data storage requirements.

Informed consent documents will be retained in locked filing cabinets and storerooms, accessible only to senior investigators and designated study staff. All questionnaires and samples are de-identified at the time of collection. The lists linking study numbers to names are kept separately on a password-protected computer, accessible only to the PI, Ugandan PI, and project manager. Standard service-related clinical and HIV counseling records and consents are kept in secure files in locked offices, accessed only by approved personnel. Clinicians and counselors have received training on the need to maintain complete confidentiality of client data. All computerized databases and files will contain only study ID numbers.

### Data analysis

#### Interim analyses

Interim analysis will be conducted after 50% of the study participants have completed their 12-month evaluations, with the following objectives: (1) examine the validity of assumptions made for estimating sample size; (2) decide whether early termination of the trial is warranted due to clear efficacy or lack thereof; and (3) determine whether there are unanticipated reasons for early trial termination or modification. No stopping rules have been predefined for this trial, given that the research procedures are minimally invasive and present low risks to the study participants.

#### Statistical methods for primary outcomes

The primary outcome of the trial is viral suppression, defined as a plasma HIV-1 RNA concentration < 200 copies/mL [38]. An intent-to-treat analysis will be used to assess the primary outcome at 12 months among PLWH in the intervention group compared with the control group. A mixed effects logistic regression model—with individual PLWH in level 1 nested within TH in level 2—will be applied to determine if the intervention increases the relative risk of viral load suppression compared with the control. We will conduct additional analyses to account for the heterogeneous effect of individual PLWH-level parameters on viral suppression in the mixed effects logistic regression model. The individual PLWH-level parameters to be accounted for may include newly diagnosed patients with HIV at enrollment vs. previously diagnosed patients; demographic variables such as age, sex, and socioeconomic status; and survey measures such as social support and HIV stigma. We will also apply an estimand framework analysis to consider the impact of intercurrent events on the primary outcome of HIV viral suppression. *p* < 0.05 will considered to indicate statistical significance in this primary analysis. Participants lost to follow-up will be considered virally unsuppressed for the primary outcome analysis.

#### Statistical methods for secondary outcomes

Secondary analyses based on *p* values adjusted for multiple comparisons included fitting regression models to determine the impact of the intervention on linkage to care within 7 days, ART initiation, ART adherence, and retention in care at 12 months. To evaluate ART adherence, we will compare the binary outcomes and continuous values for hair tenofovir concentration between study arms. The effect of the intervention on quality of life will be assessed using mixed effects linear regression by comparing the intervention and control groups. We will also evaluate changes in quality of life measured over 12 months by paired *t*-test. Finally, we will evaluate sex as a biological variable to assess whether the intervention’s effect differs between men and women.

For the implementation evaluation, quantitative analysis will proceed with a descriptive statistical analysis for continuous variables such as the TFA and fidelity scores. Outcomes will be compared across TH and HIV clinician sociodemographic characteristics using logistic regression and mixed linear models. Mixed effects logistic regression models will be used to assess intervention maintenance using HIV viral load in blood plasma samples.

#### Qualitative and mixed methods analysis

Qualitative data analysis will incorporate a priori codes from CFIR domains to generate initial codes. The iterative nature of data collection will also allow for the inclusion of emerging codes in the codebook. English transcripts will be read and coded by the Qualitative Data Coordinator and US principal investigator. Codes will be harmonized, reviewed, organized into themes, and examined to identify patterns pertinent to implementation constructs. Collaborators will review the data twice per month to determine, through consensus, when theoretical saturation has been reached. NViVo 11 (QSR International Pty Ltd.) will be used for data organization.

A convergent mixed methods approach will assign equal weight to qualitative and quantitative data to evaluate aspects of intervention implementation according to the domains in Table 2. We will evaluate qualitative and quantitative datasets to identify areas of convergence and discordance [71]. Trends in the survey data regarding implementation context and outcomes will be examined and merged with pertinent qualitative findings using a joint display approach [72]. The data will be triangulated to investigate various variable relationships, for example, correlations between PLWH, TH, and HIV clinicians’ attitudes toward viral load suppression or how qualities of internal settings align with successful collaborations between TH and HIV clinicians.

### Oversight and monitoring

The study has an Administrative Core, Steering Committee, and Data Safety and Monitoring Board (DSMB). The Administrative Core comprises the study principal investigators, a full-time Ugandan project manager, and a full-time US research coordinator. Together, they oversee trial activities and regulatory requirements and coordinate team meetings. The Steering Committee comprises principal investigators, coinvestigators, a project manager, quantitative and qualitative data managers, and two Ugandan research assistants. This committee ensures adherence to the study protocol and standard operating procedures to achieve the stated research goals.

The three-person DSMB is an independent external monitoring committee that includes a biostatistician, clinical trialist, and HIV clinician. The DSMB met prior to study initiation to review the consent forms and protocol and will meet every 6 months throughout the study period or more frequently as needed. Prior to their meeting, the DSMB will receive open reports of study progress and all adverse events (please see below), including breaches of confidentiality, stigma, and social harm. After the 325th participant has been enrolled, the DSMB will be presented with interim analysis results and decide whether to terminate, modify, or continue the trial.

### Adverse event reporting and harms

An adverse event (AE) is defined as any untoward medical occurrence in a participant regardless of the possibility of a causal relationship to trial participation [73]. All adverse events occurring after the point of enrollment will be collected systematically from each participant. At each study visit, participants will be asked whether they “have experienced any problems as a result of being in this study.” All AEs reported after enrollment and through the end of the study period will be defined and graded for severity based on guidance from the Health and Human Services Office of Human Research Protections [73]. The suspected relationship to trial intervention will be determined by the principal investigators and documented along with the interventions given and the outcome. This documentation will be reported to the local Institutional Review Board (IRB), the DSMB, and the trial sponsor.

A serious adverse event (SAE) is any event that is fatal or life-threatening, results in persistent or significant disability or incapacity, constitutes a congenital anomaly or disability, or requires inpatient hospitalization or prolongation of existing hospitalization [73]. An SAE may also include an event that is sufficiently significant to require medical or surgical intervention to prevent one of these serious outcomes or is a hazard determined by the DSMB. An AE that meets the criteria for an SAE between enrollment and the end of the study will be reported to the local IRB, the DSMB, and the trial sponsor.

The decision for study discontinuation of a study participant will be made by the principal investigators and/or the study participant. Study personnel will document the circumstances and data leading to discontinuation. The principal investigators will receive a weekly log of any AEs and will review this log each week with the Administrative Core.

### Plans for communicating important protocol amendments to relevant parties

The Mbarara University of Science and Technology Ethics Committee and Weill Cornell Medicine IRB will be notified of all protocol amendments as per regulations provided by the respective parties. Trial participants will be notified by telephone of changes to the trial protocol and will be reconsented if necessary.

### Dissemination plans

The trial has been registered on ClinicalTrials.gov, and the record will be updated at least once per year and within 30 days of a change to applicable sections of the ClinicalTrials.gov record. Final trial results will be posted on ClinicalTrials.gov no later than 1 year after the study’s primary completion date. Preliminary results will be presented twice per year to our ten-person traditional healer Community Advisory Board and to the local academic community at the annual Mbarara University of Science and Technology Research Dissemination Conference. In addition, we will organize annual dissemination meetings with officers from the Ugandan Ministry of Health AIDS Control and Community Health Divisions, along with stakeholders from civil society organizations. Scientific abstracts will be submitted to international and domestic conferences, and trial results will be submitted for publication in peer-reviewed journals, with all publications as open access.

## Discussion

While there is observational evidence suggesting that TH can be leveraged as lay providers to improve engagement in HIV care, there is a dearth of systematic trials addressing this subject [34, 39, 74–76]. This clinical trial is essential for definitively evaluating the impact of TH-supported care on HIV suppression in rural communities and optimizing the allocation of programmatic resources to low-income contexts. The implementation science approach employed in this study can accelerate evidence into practice by collecting data pertinent to intervention effectiveness concurrently with data on the implementation context, facilitators and barriers to program success. If successful, this approach could transform HIV programming in rural regions where PLWH face numerous barriers to entering and remaining with HIV and support progress toward the UNAIDS 2030 goals to end the HIV epidemic [5].

There are some limitations to this study, which include that it is being evaluated in a geographically limited area of rural Uganda. However, we designed the implementation evaluation of this study using the CFIR to rigorously evaluate the contextual drivers affecting the delivery and effectiveness of the intervention. These types of data have been successfully applied to guide adaptations and delivery of numerous evidence-based programs, and we anticipate a similar ability to generalize findings to improve HIV outcomes across Uganda and other African countries where TH are ubiquitous members of rural communities [31, 77, 78].

### Trial status

Protocol 2.0 version date: September 29, 2023.

Date recruitment began: July 7, 2023.

Approximate date of recruitment completion: July 30, 2025.

### Supplementary Information

Additional file 1: SPIRIT checklist for *Trials*. This document is required by *Trials* to submit alongside a study protocol submission. This SPIRIT checklist provides guidance on what information is included in this study protocol submission and where in the submission document the information is located.

## Data Availability

The final de-identified dataset will be available upon request made to the first author and following the completion of a data-sharing agreement.

## Abbreviations

AE: Adverse event
ART: Antiretroviral therapy
CFIR: Consolidated Framework for Implementation Research
DSMB: Data Safety and Monitoring Board
HIV: Human immunodeficiency virus
IRB: Internal Review Board
PLWH: People living with HIV
SAE: Serious adverse event
TH: Traditional healer
